# Significant Associations between *Chlamydia trachomatis* and *Neisseria gonorrhoeae* Infections in Human Immunodeficiency Virus-Infected Pregnant Women

**DOI:** 10.1155/2022/7930567

**Published:** 2022-06-17

**Authors:** Bongekile Ngobese, Khine Swe Swe-Han, Partson Tinarwo, Nathlee S. Abbai

**Affiliations:** ^1^School of Clinical Medicine Laboratory, College of Health Science, Nelson R Mandela School of Medicine, University of KwaZulu-Natal, Durban, South Africa; ^2^Department of Medical Microbiology, School of Laboratory Medicine and Medical Sciences, College of Health Sciences, Nelson R Mandela School of Medicine, University of KwaZulu-Natal, Durban, South Africa; ^3^Department of Microbiology, National Health Laboratory Services, KwaZulu-Natal Academic Complex, Inkosi Albert Luthuli Central Hospital, Durban, South Africa; ^4^Department of Biostatistics, Nelson R Mandela School of Medicine, University of KwaZulu-Natal, Durban, South Africa

## Abstract

There is a lack of data on the burden of *Chlamydia trachomatis* and *Neisseria gonorrhoeae* among human immunodeficiency virus- (HIV-) infected pregnant women in South Africa. We conducted a cross-sectional study which included 385 HIV-infected pregnant women attending antenatal clinic at the King Edward VIII Hospital in Durban, South Africa. The women provided vaginal swabs which were tested for *C. trachomatis* and *N. gonorrhoeae*. The prevalence of the individual STIs was as follows: *C. trachomatis* (47/385, 12.2%) and *N. gonorrhoeae* (16/385, 4.1%). Having a circumcised partner, testing positive for *N. gonorrhoeae*, and perceiving themselves of being at risk for infection were shown to increase the risk for *C. trachomatis* infection. Without controlling for the other factors, testing positive for *N. gonorrhoeae* increased the risk for *C. trachomatis* infection by 10-fold (OR: 10.17, 95% CI: 3.39-29.66, *p* < 0.001). Similarly, adjusting for the other factors, the risk for *C. trachomatis* infection in women who tested positive for *N. gonorrhoeae* was 9-fold (OR: 9.16, 95% CI: 2.19-40.18, *p* = 0.003). The following factors were associated with the increased risk of *N. gonorrhoeae* infection: not knowing their partner's HIV status, partner having other partners, and *C. trachomatis* infection status. Without controlling for the other factors, testing positive for *C. trachomatis* increased the risk for *N. gonorrhoeae* infection by 6-fold (OR: 6.52, 95% CI: 2.22-18.49, *p* < 0.001). Similarly, adjusting for the other factors, the risk for *N. gonorrhoeae* infection in women who tested positive for *C. trachomatis* was 6-fold (OR: 6.09, 95% CI: 1.73-22.03, *p* = 0.005). We found a significant association between *C. trachomatis* and *N. gonorrhoeae* in the pregnant women and the risk factors associated with these pathogens. Future studies are urgently required to investigate the impact of *C. trachomatis*/*N. gonorrhoeae* coinfections in HIV pregnant women since this data is lacking in our setting. In addition, etiological screening of *C. trachomatis* and *N. gonorrhoeae* during antenatal clinic is urgently required to prevent adverse pregnancy and birth outcomes associated with these infections.

## 1. Introduction

C*hlamydia trachomatis* (*C. trachomatis*) and *Neisseria gonorrhoeae* (*N. gonorrhoeae*) are the most commonly reported bacterial sexually transmitted infections (STIs) in humans worldwide, with an estimated 131 million new cases of chlamydia and 78 millions of gonorrhoea among adults in 2016 [1]. In the African region, *C. trachomatis* and *N. gonorrhoeae* account for 12 and 11.4 million new cases per year, respectively [1, 2]. The majority of new STIs occur among women aged between 15 and 49 years old [3]. South Africa has one of the highest prevalence rates of human immunodeficiency virus (HIV) and STIs, with prevalence and incidence rates higher than other African countries [4, 5]. KwaZulu-Natal (KZN), one of South Africa's most densely populated provinces, has been reported to be significantly affected by both HIV and STIs particularly among pregnant and nonpregnant young women [6]. However, the prevalence of STIs has not been well documented to date especially in HIV-infected pregnant women.

In Africa, STIs are a major cause of maternal and perinatal morbidity among HIV-infected pregnant women and are associated with adverse pregnancy and birth outcomes [7–9]. In most cases, *C. trachomatis* and *N. gonorrhoeae* infections are asymptomatic especially in pregnant women [10, 11]. According to the World Health Organization (WHO), approximately in 60% to 80% of cases, infection with *C. trachomatis* does not manifest symptoms and remains asymptomatic [1]. Similarly, studies have reported that 50% to 80% of women infected with *C. trachomatis* do not develop symptoms, and these women are considered silent reservoirs of the pathogen and continue to transmit it sexually [12]. Moreover, physiological changes during pregnancy such as changes in vaginal discharge and urinary habits may further mask the signs and symptoms of true infection [13].

*C. trachomatis* and *N. gonorrhoeae* are significant etiological agents of cervicitis, accounting for up to 50% of cases [14]. If left untreated, it can cause a variety of adverse effects and complications, such as pelvic inflammatory disease, miscarriage, premature rupture of membranes, low birth weight, and preterm labour and delivery [15–17]. During labour, these infections can be transmitted vertically from pregnant women to their newborns [18, 19]. Studies have shown that the risk of the adverse events in pregnancy and neonates is increased when coinfections with these two organisms are present [15]. Infection with *C. trachomatis* can lead to neonatal ophthalmia neonatorum and pneumonia, while infection with *N. gonorrhoeae* can cause neonatal ophthalmia neonatorum and blindness [20, 21]. Several studies have reported that risk factors associated with *C. trachomatis* and *N. gonorrhoeae* include younger age, condomless sex, being unmarried, having more than one sexual partner, being unemployed, low level education attainment, frequent alcohol use, frequent tobacco use, illegal substance use, and a history of STIs [11, 22, 23].

Coinfection with HIV and *C. trachomatis* or *N. gonorrhoeae* is associated with an increased risk of mother-to-child transmission of HIV [7]. To date in KZN, South Africa, there is limited recent data on the prevalence, coinfection rates, and risk factors for curable STIs in HIV-infected pregnant women. In this study, HIV-infected pregnant women were tested for the presence of *C. trachomatis* and *N. gonorrhoeae*. This study also assessed the actual risk versus perceived risk of contracting these STIs in the studied population.

## 2. Methods

### 2.1. Ethical Statement

Ethics approval for this study (BREC/00001382/2020) was obtained from the Biomedical Research Ethics Committee (BREC), University of KZN.

### 2.2. Study Population

This was a cross-sectional study among pregnant women, attending the antenatal clinic (ANC) at the King Edward VIII Hospital in Durban, South Africa. At the clinic, women were educated on the consequences of STIs during pregnancy and provided with information on risk reduction for STIs. The clinic attends to 80 to 100 women on a daily basis, and the recruitment for this study took place between October 2020 and April 2021. Women were enrolled in this study if they were HIV infected, 18 years and older, willing to provide written informed consent, willing to provide vaginal swab samples, and willing to provide sociodemographic, behavioral, and clinical data. Patients were explained the process of self-sample collection. Each enrolled women provided self-collected vaginal swabs (dry swabs) for detection of the vaginal infections. The consenting women had also completed a questionnaire on sociodemographic, behavioral, and clinical factors.

### 2.3. Laboratory Procedures

#### 2.3.1. Sample Processing

After collection, the dry swabs were placed in 2 ml of phosphate buffered saline (PBS). The solution was vortexed to dislodge the cells from the swabs, and the swab was discarded. The remaining suspension was centrifuged at 14,000 rpm for 10 mins, and the supernatant was discarded. Recovered pellets were then subjected to further molecular analysis.

#### 2.3.2. DNA Extraction

DNA extraction was performed on the vaginal fluid pellets using the PureLink Microbiome Kit (Thermo Fisher Scientific, United States), according to the manufacturer's instructions. Briefly, 2 ml of the vaginal fluid samples was centrifuged for 30 minutes at 14 000 × *g*. The supernatant was discarded, and 800 *μ*l of S1 lysis buffer was added to the pellet and pipetted up and down to mix the sample. The sample was then transferred to the bead tube, and 100 *μ*l of S2 lysis enhancer was added to the bead tube, capped, and vortexed briefly. This was incubated at 95°C for 10 minutes, followed by vortexing at a maximum speed for 7 minutes and further centrifuged at 14 000 × *g* for 1 minute. Thereafter, 500 *μ*l of the supernatant was transferred to a clean microcentrifuge tube, avoiding the bead pellet and any cell debris. To bind DNA to the column, 900 *μ*l of binding buffer was added and vortexed briefly. Following this, 700 *μ*l of the sample mixture was loaded onto a spin column-tube and centrifuged at 14 000 × *g* for 1 minute. The flow through was discarded, and the spin column was centrifuged at 14 000 × *g* for 30 seconds. The spin column was placed in a clean tube; 50 *μ*l of S6 elution buffer was added, and the tube was incubated at room temperature for 1 minute. After 1 minute, the spin column was centrifuged at 14 000 × *g* for 1 minute, and the column was discarded, and the purified DNA was stored at -20°C. The concentration of extracted DNA was determined using the NanoDrop spectrophotometer (Thermo Fisher Scientific, United States). DNA samples were stored at –20°C until further molecular analysis. The molecular assays were conducted at the School of Clinical Medicine's Research Laboratory at the University of KZN.

### 2.4. Detection of STI Pathogens

PCR amplification was performed on the Quant Studio 5 real-time PCR detection system (Thermo Fisher Scientific, Waltham, Massachusetts, United States of America), in a 96-well microtiter reaction plate. We screened for *C. trachomatis* and *N. gonorrhoeae* using the Applied Biosystems™ TaqMan® Assays. The following commercial primers and probes (Ba04646249_S1 and Ba04646252_S1; Thermo Fisher Scientific, Waltham, Massachusetts, United States of America) were used for each organism, respectively. The Ba04646249_S1 targets the translocated actin-recruiting phosphoprotein gene of *C. trachomatis*, and Ba04646252_S1 targets the hypothetical protein gene of *N. gonorrhoeae*. Each PCR reaction was performed in a final volume of 20 *μ*l comprising 1 *μ*l FAM-labeled probe/primer mix, 5 *μ*l Fast Start 4x probe master mix (Thermo Fisher, Part No. 4444434), 2 *μ*l template DNA, and 11 *μ*l nuclease-free water. Nontemplate and positive controls (TaqMan™ Vaginal Microbiota Extraction Control; cat no. A32039) were also included. Amplification was performed at 95°C for 30 seconds followed by 45 cycles comprising of denaturation at 95°C for 3 seconds and annealing at 60°C for 30 seconds. Detection of amplified fluorescent products was carried out at the end of the annealing phase. The raw fluorescent data that included the *C*_*T*_ mean values were automatically generated by the Quant Studio 5 Real-time PCR system software.

### 2.5. Data Analyses

The statistical data analysis was conducted in the R Statistical computing software of the R Core Team, 2020, version 3.6.3. The descriptive statistics of numerical measurements were summarized as the minimum, maximum, quartiles, interquartile range, means, standard deviation, and the coefficient of variation. On the other hand, the categorical variables were described as counts and percentage frequencies. Depending on the distribution of the numerical variables between two independent groups, mean or median differences were assessed using either *t*-test or Wilcoxon test, respectively. To determine the association between categorical variables, a chi-square test was used, and when the distribution of the cross tabulations contained an expected value of less than five, a Fisher's exact test was applied. Binary logistic regression was used to determine the factors associated with infection and presented as odds ratios. All the inferential statistical analysis tests were conducted at 5% levels of significance.

## 3. Results

### 3.1. Prevalence Estimates and Actual Risk versus Perceived Risk

Of the total 385 HIV-infected women who were enrolled in this study, the prevalence of the individual STIs was as follows: *C. trachomatis* (47/385, 12.2%) and *N. gonorrhoeae* (16/385, 4.1%) (Tables 1 and 2). The Ct values of the samples are shown in Supplementary Table S1. According to the Ct values, using a cut-off value of 25 for distinguishing low and high positives (<25 high positive, >25 low positive), 43 samples were low positive,s and four samples were high positives were *C. trachomatis*, and 16 samples were low positives, and none of the samples were high positives were *N. gonorrhoeae*.

For the women who tested negative for *C. trachomatis*, 31.9% reported that they were not at risk for contracting an STI. The majority of women (68.1%) who perceived themselves for being at risk for infection tested positive. However, there was no significant association between risk perception and infection status for *C. trachomatis* (*p* = 0.274; Table 1).

For *N. gonorrhoeae*, a similar trend was observed; a larger percentage of the women (68.8%) who perceived themselves at being at risk for contracting the STI tested positive. Despite the high percentage of women being at actual risk, there was no significance (*p* = 0.505) between actual versus perceived risk for this pathogen (Table 2).

### 3.2. Characteristics of the Study Women according to *C. trachomatis* Status

There was a significant difference (*p* < 0.001) in age across the women who tested *C. trachomatis* negative and positive. The women who tested positive were younger (median age: 26.0; interquartile range: 21.5-32.0) when compared to the women who tested negative (median age: 31.0; interquartile range: 26.0-37.0). Factors significantly associated with *C. trachomatis* status included marital status, age of first sex, partner being circumcised, previous treatment for STIs, and *N. gonorrhoeae* status. A higher proportion of unmarried women (97.9%) tested positive for *C. trachomatis* when compared to 85.8% who tested negative (*p* = 0.020). A higher proportion of women who had experienced first sex younger than 15 years of age tested *C. trachomatis* positive (10.6%) when compared to 2.1% who tested negative for infection (*p* = 0.009). Having a circumcised partner does not protect against infection. For the women who reported having a circumcised partner, a lower proportion tested negative for infection (66.0%) when compared to 80.9% who tested *C. trachomatis* positive (*p* = 0.041). Having a history of STI treatment protected against infection showing that 35.5% tested negative for infection when compared to 19.1% who tested positive (*p* = 0.026). A higher proportion (14.9%) of women who tested positive for *N. gonorrhoeae* also tested positive for *C. trachomatis* when compared to 2.7% who tested negative (*p* = 0.001; Table 1).

### 3.3. Characteristics of the Study Women Who Tested *N. gonorrhoeae* Positive

There was a significant difference (*p* = 0.004) in age across the women who tested *N. gonorrhoeae* negative and positive. The women who tested positive were younger (median age: 24.5; interquartile range: 21.8-29.3) when compared to the women who tested negative (median age: 30.0; interquartile range: 25.0-36.0). Other factors significantly associated with *N. gonorrhoeae* status included partner's HIV status, partner having other partners, and *C. trachomatis* status. A higher proportion of women who did not know their partner's HIV status tested positive for *N. gonorrhoeae* (43.8%) when compared to 14.1% who tested negative for infection (*p* = 0.006). A higher proportion of women who reported that their partner had other partners tested positive for *N. gonorrhoeae* (50.0%) when compared to 23.0% who tested negative (*p* = 0.026). Of the women who tested *C. trachomatis* positive, a higher proportion (43.8%) tested positive for *N. gonorrhoeae* when compared to 10.8% who tested negative (*p* = 0.001; Table 2).

### 3.4. Risk Factors for *C. trachomatis* Infection

The following factors were associated with the increased risk of acquiring *C. trachomatis* infection: partner being circumcised, perceiving one's self at being at risk for infection, and *N. gonorrhoeae* infection status (Table 3).

Having a circumcised partner, testing positive for *N. gonorrhoeae*, and perceiving themselves of being at risk for infection were shown to increase the risk for *C. trachomatis* infection. In the unadjusted analysis, having a circumcised partner increased the risk for infection by 5-fold (odds ratio (OR): 5.03, 95% confidence interval (CI): 1.74-21.32, *p* = 0.009). After controlling for confounders, having a circumcised partner increased the risk for infection by close to 4-fold (OR: 3.86, 95% CI: 1.21-17.27, *p* = 0.039). Perceiving one's self to be at risk for infection was shown to increase the risk for infection by greater than 2.5-fold in the adjusted analysis only (OR: 2.85, 95% CI: 1.16-7.68, *p* = 0.028). Testing positive for *N. gonorrhoeae* increased the risk for *C. trachomatis* infection by 10-fold in the unadjusted analysis (OR: 10.17, 95% CI: 3.39-29.66, *p* < 0.001). Similarly, after controlling for confounders, the risk for *C. trachomatis* infection in women who tested positive for *N. gonorrhoeae* was 9-fold (OR: 9.16, 95% CI: 2.19-40.18, *p* = 0.003).

However, in the unadjusted analysis, having sex at an older age, i.e., older than 21 years of age, reduced the risk of getting infected with *C. trachomatis* (OR: 0.03, 95% CI: 0.00-0.20, *p* < 0.001). This association was also shown to be significant in the adjusted analysis (OR: 0.08, 95% CI: 0.01-0.63, *p* = 0.020). Having been previously treated for STIs also reduced the risk for *C. trachomatis* infection, both in the unadjusted and adjusted analysis (OR: 0.19, 95% CI: 0.04-0.54, *p* = 0.006, and OR: 0.16, 95% CI: 0.03-0.51, *p* = 0.006, respectively). In the unadjusted analysis, cohabiting with a partner also reduced the risk of getting infected with *C. trachomatis* (OR: 0.32, 95% CI: 0.12-0.76, *p* = 0.015) (Table 3).

### 3.5. Risk Factors for *N. gonorrhoeae* Infection

The following factors were associated with the increased risk of acquiring *N. gonorrhoeae* infection: not knowing their partner's HIV status, partner having other partners, engaging in intravaginal practices, and being *C. trachomatis* positive (Table 4).

In the unadjusted analysis, not knowing their partner's HIV status increased the risk for infection by 4-fold (OR: 4.75, 95% CI: 1.38-28.71, *p* = 0.016). Similarly, in the adjusted analysis, not knowing their partner's HIV status increased the risk for infection by 5-fold (OR: 5.73, 95% CI: 1.35-28.71, *p* = 0.022), and this was significant. In the adjusted analysis, women who reported that their partner had other partners had a close to 4-fold increased risk of getting infected, and this was significant (OR: 3.91, 95% CI: 1.13-14.65, *p* = 0.034). Engaging in intravaginal practices increased the risk of infection by 5-fold (OR: 5.83, 95% CI: 0.76-31.51, *p* = 0.052) in the adjusted analysis. Testing positive for *C. trachomatis* increased the risk for *N. gonorrhoeae* infection by 6-fold in the unadjusted analysis (OR: 6.52, 95% CI: 2.22-18.49, *p* < 0.001). Similarly, in the adjusted analysis, the risk for *N. gonorrhoeae* infection in women who tested positive *C. trachomatis* increased by 6-fold (OR: 6.09, 95% CI: 1.73-22.03, *p* = 0.005), and this association was significant. However, in the adjusted analysis, being in the second and third trimesters of pregnancy reduced the risk of getting infected with *N. gonorrhoeae* (odds ratio (OR): 0.16, 95% confidence interval (95% CI): 0.03-1.03, *p* = 0.045, and OR: 0.13, 95% CI: 0.02-0.76, *p* = 0.016, respectively) Table 4).

## 4. Discussion

In this study, the prevalence of investigated STIs was as follows: *C. trachomatis*, 12.2%, and *N. gonorrhoeae*, 4.1%. The majority of the samples were low positives for both pathogens. This is expected since these pathogens are cervical pathogens, and the sample analyzed was from the vagina which has a lower abundance of *C. trachomatis* and *N. gonorrhoeae.* In the current study of the women who tested positive for *C. trachomatis*, 14.9% tested positive for *N. gonorrhoeae*, and of the women who tested positive for *N. gonorrhoeae*, 43.8% tested for *C. trachomatis* (*p* < 0.001). A study conducted among HIV-infected and HIV-uninfected pregnant women showed a *C. trachomatis/N. gonorrhoeae* coinfection rate of 7% in HIV-infected women [10]. Similarly, in a study by Smullin et al., a *C. trachomatis*/*N. gonorrhoeae* coinfection of 5.4% was reported for HIV-infected women [24], and a study by Nyemba et al. reported a coinfection rate of 7.7% for *C. trachomatis/N. gonorrhoeae* in HIV-infected women [25]. Coinfection with HIV and *C. trachomatis/N. gonorrhoeae* has been reported to be associated with risk of mother-to-child transmission of HIV [7]. Similarly, Reda et al. reported that there are clear connections between HIV transmission and infection with chlamydia and gonococcus [26]. Furthermore, several studies have reported that pregnant women with STIs have twice risk of giving birth to an HIV-infected infant, particularly in women with two STIs [7, 15, 27].

Of the women who perceived themselves for not being at risk for contracting STIs, 48.3% tested positive for at least one STI. This study highlighted that a woman's perceived risk and her actual risk may differ. This study identified risk factors associated with STI acquisition with the majority of the factors being linked to the women's as well as their partners' behavior. Therefore, in order to implement behavioral interventions to reduce HIV/STI risk, we will need to take into consideration the woman's and her partner's sexual behavior patterns. This can be facilitated using interventions targeting couples.

The prevalence estimates for *C. trachomatis* among HIV-infected pregnant women range from 6.5% to 36.8% in South Africa. The prevalence of *C. trachomatis* infection in the current study was 12.2%. Studies conducted in Tshwane District in South Africa among HIV-infected pregnant women reported the prevalence of *C. trachomatis* infections to be between 17% and 36.8% [13, 22, 27–29]. Studies conducted in Cape Town, South Africa, among HIV-infected pregnant women reported that the prevalence of *C. trachomatis* was 6.5% and 20%, respectively [10, 24]. Similarly, another South African study, conducted among HIV-infected and uninfected pregnant women, reported a *C. trachomatis* prevalence of 15.3% in the HIV-infected and 14.3% in the HIV-uninfected women [25].

The prevalence estimates for *N. gonorrhoeae* infection among HIV-infected pregnant women range from 0% to 6.9% in South Africa. In the current study, the prevalence of *N. gonorrhoeae* infection was 4.1%. Studies conducted in Tshwane District in South Africa among HIV-infected pregnant women reported the prevalence of *N. gonorrhoeae* infections to be between 0.9% and 6.9% [13, 22, 27–29]. Studies conducted in Cape Town, South Africa, among HIV-infected and uninfected pregnant women reported *N. gonorrhoeae* prevalence rates of 1.1% and 10%, respectively [10, 24]. In a study conducted in KwaZulu-Natal, South Africa, among pregnant women, a prevalence of 6.4% was reported for *N. gonorrhoeae* [30].

The differences in prevalence estimates observed for *C. trachomatis* and *N. gonorrhoeae* between the current study and other studies may be due to different sensitivities of detection methods used, specimen types collected, study sample sizes, behavioral, sociodemographic factors, and clinical characteristics [31]. Furthermore, studies have found that the rate of infection persistence differs between pregnant and nonpregnant women, possibly due to differences in immune response caused by hormonal regulation. Pregnant women are more vulnerable to certain infections and in some cases suffer more severe consequences than nonpregnant women [32]. While there are several studies on the physiological changes during pregnancy and postpartum periods which increase HIV risk, factors that increase risky sexual behavior during pregnancy, consequently increasing the risk of HIV and STI acquisition and transmission, are not well described [33].

Numerous demographic and behavioral factors have been reported to be associated with *C. trachomatis* and *N. gonorrhoeae* infections during pregnancy [22]. In pregnant women, younger age, low socioeconomic status, multiple sex partners, being unmarried, condomless sex, not knowing their partner's HIV status, alcohol consumption, unemployment, low education attainment, physical violence, and psychological distress have been associated with *C. trachomatis* and *N. gonorrhoeae* infections [5, 12, 22, 23, 25, 27, 34].

In this study, younger age was significantly associated with testing positive for *C. trachomatis.* It has been demonstrated by several studies that young age is a risk predictor for STIs [22, 27]. There are several biological, immunological, and behavioral factors, which contribute to STI acquisition [27], such as immature ectopic tissues on the cervix and sexual behavior which contribute to the growth of this pathogen [35]. Having sex at an early age was also associated with testing positive for *C. trachomatis* in the study. This finding is similar to findings published in other studies [11]. In this study, being unmarried was associated with testing positive for *C. trachomatis*. Similarly, a study conducted by Davey et al. in a population of pregnant women, it was observed that being unmarried was associated with being diagnosed with an STI, and the most prevalent STI in that population was *C. trachomatis* [10]. A systematic review conducted by Morris et al. confirmed that partner medical circumcision reduces women's risk for *T. vaginalis*, bacterial vaginosis, and possibly genital ulcer disease [36]. However, for herpes simplex virus type 2, *C. trachomatis*, *Treponema pallidum*, and HIV, the evidence was mixed [36]. The current study did not find a protective effect of circumcision in terms of *C. trachomatis* acquisition. In the adjusted analysis conducted in this study, it was shown that having a circumcised partner increased the risk for infection by 3-fold (OR: 3.86, 95% CI: 1.21-17.27, *p* = 0.039).

The *C. trachomatis/N. gonorrhoeae* coinfection rate was 14.9% in the studied population. According to Leonard et al., an estimated 10–40% of individuals with *N. gonorrhoeae* infection are concomitantly infected with *C. trachomatis* [37]. In addition, according to the adjusted analysis, the risk for *C. trachomatis* infection in women who tested positive for *N. gonorrhoeae* was 9-fold (OR: 9.16, 95% CI: 2.19-40.18, *p* = 0.003). Coinfections of these two STIs may increase the susceptibility to long-term complications and transmissibility of these infections [38]. Additionally, *N. gonorrhoeae* shedding was higher in women with concurrent *C. trachomatis* infection than in those infected with *N. gonorrhoeae* only [39].

Perceiving one's self to be at risk for infection was shown to increase the risk for infection by 2-fold in the adjusted analysis conducted in this study (OR: 2.85, 95% CI: 1.16-7.68, *p* = 0.028). Similarly, a study conducted by Gravningen et al. reported an increase in *C. trachomatis* prevalent infections with increasing risk perception [40]. According to Clifton et al., understanding the individuals' need to access STI care services for the testing and treatment of infections, including the individuals' risk perception for getting infected, is critical for STI control [41].

Similar to *C. trachomatis*, in this study, younger age was significantly associated with testing positive for *N. gonorrhoeae*. A similar finding was reported by Connolly et al., where younger age was significantly associated (*p* = 0.01) with the risk for *N. gonorrhoeae* infection [42]. Having an HIV-positive partner was significantly associated with having a prevalent *N. gonorrhoeae* infection. A study conducted by Davey et al. found that women who reported being in a concordant HIV-positive partnership had over twice the odds of having an STI. In that same study, 37% of women did not know their partner's HIV serostatus [10]. In this study, not knowing their partners HIV status increased the risk for infection by 5-fold (OR: 5.73, 95% CI: 1.35-28.71, *p* = 0.022).

In this study, testing positive for *C. trachomatis* increased the risk for *N. gonorrhoeae* infection by 6-fold (OR: 6.09, 95% CI: 1.73-22.03, *p* = 0.005). A study conducted by Jongen et al. found that having a prevalent *C. trachomatis* infection increased the risk of an incident *N. gonorrhoeae* infection [43]. In the adjusted analysis, women who reported that their partner had other partners had a 3-fold increased risk of getting infected with *N. gonorrhoeae* (OR: 3.91, 95% CI: 1.13-14.65, *p* = 0.034). However, a study conducted by Gaffoor et al. reported that perceived male partner concurrency was not found to be significantly associated with incident STIs [44]. A later study conducted by Abbai et al. found that having a partner that has other partners was significantly associated with bacterial vaginosis infection [45].

## 5. Conclusion

This study showed a high prevalence of *C. trachomatis* and *N. gonorrhoeae* single and coinfections in HIV-infected pregnant women residing in South Africa. A large proportion of these women were asymptomatic for infection. The highly asymptomatic nature of these STIs pose a treatment challenge since South Africa uses the syndromic management approach. To date, this approach is ineffective in controlling STIs and should be replaced by test and treat programmes. Cost effective point of care (POC) tests should be evaluated for implementation in resource limited settings. Future studies are urgently required to investigate the impact of *C. trachomatis*/*N. gonorrhoeae* coinfections in HIV-pregnant women since this data is lacking in our setting.

The limitations of the study are as follow: participants were recruited from a single clinic and were not representative of the general population. Data was self-reported, and biases could have been introduced. The study only investigated the point prevalence of STIs and did not prospectively follow-up women through pregnancy to investigate incident infections or pregnancy outcomes. The strength of this study is that it fills a gap in the literature on the prevalence of *C. trachomatis* and *N. gonorrhoeae* infection among HIV-infected pregnant women in Durban, South Africa, as well as identified the risk factors associated with these pathogens.

## Figures and Tables

**Table 1:** Characteristics of the study population based on *C. trachomatis* status.

*C. trachomatis*	Negative (*N* = 338)	Positive (*N* = 47)	*p* value	Overall (*N* = 385)
Age			<0.001	
Mean ± SD (CV%)	30.8 ± 6.50 (21.1)	26.7 ± 6.27 (23.5)		30.3 ± 6.60 (21.8)
Median (Q1-Q3)	31.0 (26.0-37.0)	26.0 (21.5-32.0)		30.0 (25.0-36.0)
Min-max	18.0-44.0	18.0-39.0		18.0-44.0
Educational level			0.090	
Primary school and below	8 (2.4%)	1 (2.1%)		9 (2.3%)
High school	255 (75.4%)	42 (89.4%)		297 (77.1%)
College, university	75 (22.2%)	4 (8.5%)		79 (20.5%)
Employed			0.089	
No	249 (73.7%)	40 (85.1%)		289 (75.1%)
Yes	89 (26.3%)	7 (14.9%)		96 (24.9%)
Married			0.020	
No	290 (85.8%)	46 (97.9%)		336 (87.3%)
Yes	48 (14.2%)	1 (2.1%)		49 (12.7%)
Has a regular sex partner			0.180	
No	18 (5.3%)	5 (10.6%)		23 (6.0%)
Yes	320 (94.7%)	42 (89.4%)		362 (94.0%)
Partner's HIV status			0.174	
Negative	127 (37.6%)	21 (44.7%)		148 (38.4%)
Positive	162 (47.9%)	16 (34.0%)		178 (46.2%)
Do not know	49 (14.5%)	10 (21.3%)		59 (15.3%)
Cohabiting with sex partner			0.066	
No	196 (58.3%)	34 (72.3%)		230 (60.1%)
Yes	140 (41.7%)	13 (27.7%)		153 (39.9%)
Age of 1st sex			0.009	
< 15 yrs	7 (2.1%)	5 (10.6%)		12 (3.1%)
15-20 yrs	238 (70.4%)	36 (76.6%)		274 (71.2%)
21-25 yrs	86 (25.4%)	6 (12.8%)		92 (23.9%)
> 25 yrs	7 (2.1%)	0 (0.0%)		7 (1.8%)
Lifetime number of sex partners			0.683	
1	96 (28.4%)	11 (23.4%)		107 (27.8%)
2-4	165 (48.8%)	26 (55.3%)		191 (49.6%)
> 4	77 (22.8%)	10 (21.3%)		87 (22.6%)
Partner has other partners			0.565	
Do not know	159 (47.0%)	26 (55.3%)		185 (48.1%)
No	96 (28.4%)	11 (23.4%)		107 (27.8%)
Yes	83 (24.6%)	10 (21.3%)		93 (24.2%)
Condom used during last sex			0.464	
No	219 (64.8%)	33 (70.2%)		252 (65.5%)
Yes	119 (35.2%)	14 (29.8%)		133 (34.5%)
Partner is circumcised			0.041	
No	115 (34.0%)	9 (19.1%)		124 (32.2%)
Yes	223 (66.0%)	38 (80.9%)		261 (67.8%)
Trimester of pregnancy			0.511	
1st	24 (7.1%)	5 (10.9%)		29 (7.5%)
2nd	110 (32.5%)	12 (26.1%)		122 (31.8%)
3rd	204 (60.4%)	29 (63.0%)		233 (60.7%)
Previously treatment for STIs			0.026	
No	218 (64.5%)	38 (80.9%)		256 (66.5%)
Yes	120 (35.5%)	9 (19.1%)		129 (33.5%)
Engages in intravaginal practices			1.000	
No	317 (93.8%)	45 (95.7%)		362 (94.0%)
Yes	21 (6.2%)	2 (4.3%)		23 (6.0%)
Perceived risk of getting STIs			0.274	
No	136 (40.2%)	15 (31.9%)		151 (39.2%)
Yes	202 (59.8%)	32 (68.1%)		234 (60.8%)
Partner STI symptoms			0.933	
No	275 (81.4%)	38 (80.9%)		313 (81.3%)
Yes	63 (18.6%)	9 (19.1%)		72 (18.7%)
Current symptoms of STI			0.754	
No	215 (63.6%)	31 (66.0%)		246 (63.9%)
Yes	123 (34.0%)	16 (34.0%)		139 (36.1%)
*N. gonorrhoeae* status			0.001	
Negative	329 (97.3%)	40 (85.1%)		369 (95.8%)
Positive	9 (2.7%)	7 (14.9%)		16 (4.2%)

**Table 2:** Characteristics of the study population based on *N. gonorrhoeae* status.

*N. gonorrhoeae*	Negative (*N* = 369)	Positive (*N* = 16)	*p* value	Overall (*N* = 385)
Age			0.004	
Mean ± SD (CV%)	30.5 ± 6.60 (21.6)	25.8 ± 4.80 (18.6)		30.3 ± 6.60 (21.8)
Median(Q1-Q3)	30.0 (25.0-36.0)	24.5 (21.8-29.3)		30.0 (25.0-36.0)
Min-max	18.0-44.0	19.0-34.0		18.0-44.0
Educational level			0.687	
Primary school and below	9 (2.4%)	0 (0.0%)		9 (2.3%)
High school	283 (76.7%)	14 (87.5%)		297 (77.1%)
College, university	77 (20.9%)	2 (12.5%)		79 (20.5%)
Employed			1.000	
No	277 (75.1%)	12 (75.0%)		289 (75.1%)
Yes	92 (24.9%)	4 (25.0%)		96 (24.9%)
Married			1.000	
No	322 (87.3%)	14 (87.5%)		336 (87.3%)
Yes	47 (12.7%)	2 (12.5%)		49 (12.7%)
Has a regular sex partner			0.612	
No	23 (6.2%)	0 (0.0%)		23 (6.0%)
Yes	346 (93.8%)	16 (100.0%)		362 (94.0%)
Partner's HIV status			0.006	
Negative	144 (39.0%)	4 (25.0%)		148 (38.4%)
Positive	173 (46.9%)	5 (31.3%)		178 (46.2%)
Do not know	52 (14.1%)	7 (43.8%)		59 (15.3%)
Cohabiting with sex partner			0.212	
No	218 (59.4%)	12 (75.0%)		230 (60.1%)
Yes	149 (40.6%)	4 (25.0%)		153 (39.9%)
Age of 1st sex			0.185	
< 15 yrs	11 (3.0%)	1 (6.2%)		12 (3.1%)
15-20 yrs	262 (71.0%)	12 (75.0%)		274 (71.2%)
21-25 yrs	90 (24.4%)	2 (12.5%)		92 (23.9%)
> 25 yrs	6 (1.6%)	1 (6.2%)		7 (1.8%)
Lifetime number of sex partners			0.794	
1	104 (28.2%)	3 (18.8%)		107 (27.8%)
2-4	182 (49.3%)	9 (56.3%)		191 (49.6%)
> 4	83 (22.5%)	4 (25.0%)		87 (22.6%)
Partner has other partners			0.026	
Do not know	178 (48.2%)	7 (43.8%)		185 (48.1%)
No	106 (28.7%)	1 (6.2%)		107 (27.8%)
Yes	85 (23.0%)	8 (50.0%)		93 (24.2%)
Condom used during last sex			0.175	
No	239 (64.8%)	13 (81.3%)		252 (65.5%)
Yes	130 (35.2%)	3 (18.8%)		133 (34.5%)
Partner circumcised			0.239	
No	121 (32.8%)	3 (18.8%)		124 (32.2%)
Yes	248 (67.2%)	13 (81.3%)		261 (67.8%)
Trimester pregnancy			0.216	
1st	26 (7.1%)	3 (18.8%)		29 (7.5%)
2nd	118 (32.1%)	4 (25.0%)		122 (31.8%)
3rd	224 (60.9%)	9 (56.3%)		233 (60.7%)
Previously treatment for STIs			0.845	
No	245 (66.4%)	11 (68.8%)		256 (66.5%)
Yes	124 (33.6%)	5 (31.3%)		129 (33.5%)
Engages in intravaginal practices			0.246	
No	348 (94.3%)	14 (87.5%)		362 (94.0%)
Yes	21 (5.7%)	2 (12.5%)		23 (6.0%)
Partner has STI symptoms			0.325	
No	298 (80.8%)	15 (93.8%)		313 (81.3%)
Yes	71 (19.2%)	1 (6.2%)		72 (18.7%)
Current symptoms of STI			0.680	
No	235 (63.7%)	11 (68.8%)		246 (63.9%)
Yes	134 (36.3%)	5 (31.3%)		139 (36.1%)
*C. trachomatis* status			0.001	
Negative	329 (89.2%)	9 (56.3%)		338 (87.8%)
Positive	40 (10.8%)	7 (43.8%)		47 (12.2%)
Perceived risk of getting STIs			0.505	
No	146 (39.6%)	5 (31.3%)		151 (39.2%)
Yes	223 (60.4%)	11 (68.8%)		234 (60.8%)

**Table 3:** Risk factors for *C. trachomatis* infection.

Factor	Unadjusted analysis Odds ratio with 95% confidence intervals	Full adjusted analysis Odds ratio with 95% confidence intervals	Backstep analysis Odds ratio with 95% confidence intervals
Age	0.86 (0.80-0.91, *p* < 0.001)	0.91 (0.84-0.99, *p* = 0.041)	0.91 (0.84-0.98, *p* = 0.020)
Being employed	0.29 (0.07-0.83, *p* = 0.044)	0.19 (0.03-0.79, *p* = 0.040)	0.30 (0.06-1.04, *p* = 0.085)
Having a regular sex partner	0.52 (0.16-2.30, *p* = 0.312)	0.91 (0.19-5.61, *p* = 0.909)	—
Having an HIV-positive partner	0.71 (0.31-1.62, *p* = 0.417)	1.21 (0.42-3.47, *p* = 0.722)	—
Not knowing partner's HIV status	1.37 (0.49-3.56, *p* = 0.524)	1.05 (0.26-3.83, *p* = 0.946)	—
Cohabiting with partner	0.32 (0.12-0.76, *p* = 0.015)	0.90 (0.25-2.99, *p* = 0.865)	—
Age of 1st sex, 15-20 yrs	0.16 (0.04-0.68, *p* = 0.008)	0.26 (0.05-1.44, *p* = 0.112)	0.23 (0.05-1.24, *p* = 0.072)
Age of 1st sex, 21+ yrs	0.03 (0.00-0.20, *p* < 0.001)	0.09 (0.01-0.78, *p* = 0.035)	0.08 (0.01-0.63, *p* = 0.020)
Having 2-4 lifetime number of sex partners	1.67 (0.71-4.39, *p* = 0.261)	2.69 (0.88-9.37, *p* = 0.098)	—
Having >4 lifetime sex partners	0.90 (0.26-2.94, *p* = 0.865)	1.25 (0.24-6.09, *p* = 0.787)	—
Partner does not have other partners	0.42 (0.13-1.07, *p* = 0.089)	0.40 (0.10-1.34, *p* = 0.155)	—
Partner has other partners	0.67 (0.25-1.57, *p* = 0.377)	0.32 (0.08-1.04, *p* = 0.073)	—
Used a condom during last	0.62 (0.25-1.36, *p* = 0.253)	0.50 (0.16-1.44, *p* = 0.219)	—
Having a partner who is circumcised	5.03 (1.74-21.32, *p* = 0.009)	4.28 (1.27-20.05, *p* = 0.033)	3.86 (1.21-17.27, *p* = 0.039)
Trimester pregnancy 2nd	0.65 (0.18-3.11, *p* = 0.547)	1.31 (0.24-9.36, *p* = 0.769)	—
Trimester pregnancy 3rd	0.79 (0.25-3.53, *p* = 0.722)	1.32 (0.25-9.26, *p* = 0.758)	—
Having been previously treated for STIs	0.19 (0.04-0.54, *p* = 0.006)	0.12 (0.02-0.44, *p* = 0.004)	0.16 (0.03-0.51, *p* = 0.006)
Engages in intravaginal practices	0.48 (0.03-2.44, *p* = 0.485)	0.23 (0.01-2.30, *p* = 0.298)	—
Perceived one's self at risk for STIs	2.01 (0.91-4.91, *p* = 0.098)	2.41 (0.89-7.14, *p* = 0.094)	2.85 (1.16-7.68, *p* = 0.028)
Being *N. gonorrhoeae* positive	10.17 (3.39-29.66, *p* < 0.001)	11.93 (2.60-60.62, *p* = 0.002)	9.16 (2.19-40.18, *p* = 0.003)

**Table 4:** Risk factors for *N. gonorrhoeae* infection.

Factor	Unadjusted analysis Odds ratio with 95% confidence intervals	Full adjusted analysis Odds ratio with 95% confidence intervals	Backstep analysis Odds ratio with 95% confidence intervals
Age	0.89 (0.81-0.96, *p* = 0.007)	0.84 (0.72-0.95, *p* = 0.011)	0.89 (0.80-0.98, *p* = 0.023)
Being employed	0.99 (0.27-2.93, *p* = 0.990)	1.58 (0.30-7.22, *p* = 0.566)	—
Married yes	0.97 (0.15-3.62, *p* = 990)	2.09 (0.16-21.64, *p* = 0.544)	—
Having an HIV-positive partner	1.02 (0.26-4.18, *p* = 0.978)	1.80 (0.38-9.51, *p* = 0.463)	1.79 (0.41-8.78, *p* = 0.445)
Not knowing partner's HIV status	4.75 (1.38-18.74, *p* = 0.016)	6.72 (1.44-37.95, *p* = 0.019)	5.73(1.35-28.71, *p* = 0.022)
Cohabiting with partner	0.49 (0.13-1.42, *p* = 0.218)	1.33 (0.23-6.68, *p* = 0.734)	—
Age of 1st sex, 15-20 yrs	0.46 (0.08-8.81, *p* = 0.478)	1.05 (0.11-28.79, *p* = 0.971)	—
Age of 1st sex, 21+ yrs	0.31 (0.04-6.64, *p* = 0.333)	1.80 (0.09-70.09, *p* = 0.715)	—
Having 2-4 lifetime number of sex partners	1.73 (0.50-7.94, *p* = 0.417)	1.65 (0.37-9.34, *p* = 0.531)	—
Having >4 lifetime sex partners	1.69 (0.36-8.78, *p* = 0.499)	1.65 (0.23-12.77, *p* = 0.616)	—
Partner does not have other partners	0.24 (0.01-1.37, *p* = 0.184)	0.11 (0.00-1.13, *p* = 0.114)	0.14 (0.01-1.07, *p* = 0.106)
Partner has other partners	2.37 (0.82-6.96, *p* = 0.107)	3.67 (0.95-15.58, *p* = 0.064)	3.91 (1.13-14.65, *p* = 0.034)
Used a condom during last	0.43 (0.10-1.36, *p* = 0.193)	0.47 (0.08-1.97, *p* = 0.338)	—
Having a partner who is circumcised	2.14 (0.67-9.46, *p* = 0.242)	1.29 (0.28-7.77, *p* = 0.762)	—
Trimester pregnancy 2nd	0.29 (0.06-1.56, *p* = 0.123)	0.15 (0.02-1.12, *p* = 0.059)	0.16 (0.03-1.03, *p* = 0.045)
Trimester pregnancy 3rd	0.35 (0.10-1.66, *p* = 0.134)	0.14 (0.02-0.90, *p* = 0.030)	0.13 (0.02-0.76, *p* = 0.016)
Having been previously treated for STIs	0.89 (0.27-2.50, *p* = 0.828)	2.33 (0.55-9.84, *p* = 0.239)	—
Engages in intravaginal practices	2.35 (0.35-9.16, *p* = 0.279)	5.10 (0.61-32.40, *p* = 0.093)	5.83 (0.76-31.51, *p* = 0.052)
Perceived one's self at risk for STIs	1.44 (0.51-4.66, *p* = 0.504)	1.29 (0.33-5.74, *p* = 0.726)	—
Being C*. trachomatis* positive	6.52 (2.22-18.49, *p* < 0.001)	7.95 (1.90-36.62, *p* = 0.005)	6.09 (1.73-22.03, *p* = 0.005)

## Data Availability

Data will be available upon request.

## Supplementary Materials

Supplementary Materials That shows the raw data of Ct values for samples testing positive for *C. trachomatis* and *N. gonorrhoeae*.

## References

[1] World Health Organization (2016). Global health sector strategy on sexually transmitted infections 2016-2021: toward ending STIs. *In Global health sector strategy on sexually transmitted infections 2016-2021: toward ending STIs*.

[2] Unemo M., Bradshaw C. S., Hocking J. S. (2017). Sexually transmitted infections: challenges ahead. *The Lancet Infectious Diseases*.

[3] Rowley J., Vander Hoorn S., Korenromp E. (2019). Chlamydia, gonorrhoea, trichomoniasis and syphilis: global prevalence and incidence estimates, 2016. *Bulletin of the World Health Organization*.

[4] Kharsany A. B., Cawood C., Lewis L. (2019). Trends in HIV prevention, treatment, and incidence in a hyperendemic area of KwaZulu-Natal, South Africa. *JAMA Network Open*.

[5] Naidoo S., Wand H., Abbai N. S., Ramjee G. (2014). High prevalence and incidence of sexually transmitted infections among women living in Kwazulu-Natal, South Africa. *AIDS Research and Therapy*.

[6] Kularatne R., Radebe F., Kufa-Chakezha T., Mbulawa Z., Lewis D. (2017). “Sentinel surveillance of sexually transmitted infection syndrome aetiologies and HPV genotypes among patients attending primary health care facilities in South Africa, April 2014—September 2015”, Johannesburg: Center for HIV and STIs. *National Institute for Communicable Diseases*.

[7] Adachi K., Klausner J. D., Bristow C. C. (2015). Chlamydia and gonorrhea in HIV-infected pregnant women and infant HIV transmission. *Sexually Transmitted Diseases*.

[8] Joseph Davey D. L., Shull H. I., Billings J. D., Wang D., Adachi K., Klausner J. D. (2016). Prevalence of curable sexually transmitted infections in pregnant women in low- and middle-income countries from 2010 to 2015. *A SystematicReview. Sexually Transmitted Diseases*.

[9] Schonfeld A., Feldt T., Tufa T. B. (2018). Prevalence and impact of sexually transmitted infections in pregnant women in central Ethiopia. *InternationalJournal of STD AIDS*.

[10] Joseph Davey D. L., Nyemba D. C., Gomba Y. (2019). Prevalence and correlates of sexually transmitted infections in pregnancy in HIV-infected and- uninfected women in Cape Town, South Africa. *PLoS One*.

[11] Masha S. C., Wahome E., Vaneechoutte M., Cools P., Crucitti T., Sanders E. J. (2017). High prevalence of curable sexually transmitted infections among pregnant women in a rural county hospital in Kilifi, Kenya. *PLoS One*.

[12] Azevedo M. J. N., Nunes S. D. S., Oliveira F. G., Rocha D. A. P. (2019). High prevalence of Chlamydia trachomatis in pregnant women attended at primary health care services in Amazon, Brazil. *Journal of the Institute of Tropical Mededicineof Sao Paulo*.

[13] Morikawa E., Mudau M., Olivier D. (2018). Acceptability and feasibility of integrating point-of-care diagnostic testing of sexually transmitted infections into a South African antenatal care program for HIV-infected pregnant women. *Infectious Diseasesin Obstetrics and Gynecology*.

[14] Rajabpour M., Emamie A. D., Pourmand M. R., Goodarzi N. N., Asbagh F. A., Whiley D. M. (2020). Chlamydia trachomatis, Neisseria gonorrhoeae, and Trichomonas vaginalis among women with genitourinary infection and pregnancy-related complications in Tehran: a cross-sectional study. *InternationalJournal of STD AIDS*.

[15] Adachi K., Nielsen-Saines K., Klausner J. D. (2016). Chlamydia trachomatis infection in pregnancy: the global challenge of preventing adverse pregnancy and infant outcomes in sub-Saharan Africa and Asia. *BioMed ReserachInternational*.

[16] Gadoth A., Shannon C. L., Hoff N. A. (2020). Prenatal chlamydial, gonococcal, and trichomonal screening in the Democratic Republic of Congo for case detection and management. *InternationalJournal of STD AIDS*.

[17] Olaleye A. O., Babah O. A., Osuagwu C. S., Ogunsola F. T., Afolabi B. B. (2020). Sexually transmitted infections in pregnancy - an update on Chlamydia trachomatis and Neisseria gonorrhoeae. *European Journal of Obstetrics & Gynecology and Reproductive Biology*.

[18] Honkila M., Wikström E., Renko M. (2017). Probability of vertical transmission of Chlamydia trachomatis estimated from national registry data. *Sexually Transmitted Infections*.

[19] Chang J. H., Huang Y. L., Chen C. C., Li S. Y. (2013). Vertical transmission of Neisseria gonorrhoeae to a female premature neonate with congenital pneumonia. *Journal of the Formosan Medical Association*.

[20] Beem M. O., Saxon E. M. (1977). Respiratory-tract colonization and a distinctive pneumonia syndrome in infants infected with Chlamydia trachomatis. *The New England Journal of Medicine*.

[21] Hammerschlag M. R. (2011). Chlamydial and gonococcal infections in infants and children. *Clinical Infectious Diseases*.

[22] Joseph Davey D., Peters R. P. H., Kojima N. (2018). Sexual behaviors of human immunodeficiency virus-infected pregnant women and factors associated with sexually transmitted infection in South Africa. *Sexually Transmitted Diseases*.

[23] Adachi K., Xu J., Yeganeh N. (2018). Combined evaluation of sexually transmitted infections in HIV-infected pregnant women and infant HIV transmission. *PLoS One*.

[24] Smullin C. P., Green H., Peters R. (2020). Prevalence and incidence of Mycoplasma genitalium in a cohort of HIV-infected and HIV-uninfected pregnant women in Cape Town, South Africa. *Sexually Transmitted Infections*.

[25] Nyemba D. C., Medina-Marino A., Peters R. P. H. (2021). Prevalence, incidence and associated risk factors of STIs during pregnancy in South Africa. *Sexually Transmitted Infections*.

[26] Reda S., Gonçalves F. A., Mazepa M. M., de Carvalho N. S. (2018). Women infected with HIV and the impact of associated sexually transmitted infections. *International Journal of Gynaecology and Obstetrics*.

[27] Mudau M., Peters R. P., De Vos L. (2018). High prevalence of asymptomatic sexually transmitted infections among human immunodeficiency virus-infected pregnant women in a low-income South African community. *International Journal of STD AIDS*.

[28] Peters R., Klausner J. D., de Vos L., Feucht U. D., Medina-Marino A. (2021). Aetiological testing compared with syndromic management for sexually transmitted infections in HIV-infected pregnant women in South Africa: a non- randomised prospective cohort study. *An International Journal of Obstetrics and Gynaecology*.

[29] Medina-Marino A., Mudau M., Kojima N. (2020). Persistent Chlamydia trachomatis, Neisseria gonorrhoeae or Trichomonas vaginalis positivity after treatment among human immunodeficiency virus-infected pregnant women, South Africa. *International Journal of STD AIDS*.

[30] Moodley D., Moodley P., Sebitloane M. (2015). High prevalence and incidence of asymptomatic sexually transmitted infections during pregnancy and postdelivery in KwaZulu Natal, South Africa. *Sexually Transmitted Diseases*.

[31] Zenebe M. H., Mekonnen Z., Loha E., Padalko E. (2021). Prevalence, risk factors and association with delivery outcome of curable sexually transmitted infections among pregnant women in southern Ethiopia. *PLoS One*.

[32] Juliana N. C. A., Omar A. M., Pleijster J. (2021). The natural course of Chlamydia trachomatis, Neisseria gonorrhoeae, Trichomonas vaginalis, and Mycoplasma genitalium in pregnant and post-delivery women in Pemba Island, Tanzania. *Microorganisms*.

[33] Drake A. L., Wagner A., Richardson B., John-Stewart G. (2014). Incident HIV during pregnancy and postpartum and risk of mother-to-child HIV transmission: a systematic review and meta-analysis. *PLoS Medicine*.

[34] Hoffman C. M., Mbambazela N., Sithole P. (2019). Provision of sexually transmitted infection services in a mobile clinic reveals high unmet need in remote areas of South Africa: a cross-sectional study. *SexuallyTransmitted Diseases*.

[35] Taku O., Brink A., Meiring T. L. (2021). Detection of sexually transmitted pathogens and co-infection with human papillomavirus in women residing in rural Eastern Cape, South Africa. *PeerJ*.

[36] Morris B. J., Hankins C. A., Banerjee J. (2019). Does male circumcision reduce women’s risk of sexually transmitted infections, cervical cancer, and associated conditions?. *Frontiers in Public Health*.

[37] Leonard C. A., Schoborg R. V., Low N., Unemo M., Borel N. (2019). Pathogenic interplay between Chlamydia trachomatis and Neisseria gonorrhoeae that influences management and control efforts—more questions than answers?. *Current Clinical Microbiology Reports*.

[38] Seo Y., Choi K.-H., Lee G. (2021). Characterization and trend of co-infection with Neisseria gonorrhoeae and Chlamydia trachomatis from the Korean National Infectious Diseases Surveillance Database. *The World Journal ofMen’s Health*.

[39] Stupiansky N. W., Van Der Pol B., Williams J. A., Weaver B., Taylor S. E., Fortenberry J. D. (2011). The natural history of incident gonococcal infection in adolescent women. *Sexually Transmitted Diseases*.

[40] Gravningen K., Braaten T., Schirmer H. (2016). Self-perceived risk and prevalent chlamydia infection among adolescents in Norway: a population-based cross-sectional study. *SexuallyTransmittedInfections*.

[41] Clifton S., Mercer C. H., Sonnenberg P. (2018). STI risk perception in the British population and how it relates to sexual behaviour and STI healthcare use: findings from a cross-sectional survey (Natsal-3). *eClinicalMedicine*.

[42] Connolly S., Wall K. M., Parker R. (2020). Sociodemographic factors and STIs associated with Chlamydia trachomatis and Neisseria gonorrhoeae infections in Zambian female sex workers and single mothers. *International Journal of STD AIDS*.

[43] Jongen V. W., Schim van der Loeff M. F., Botha M. H., Sudenga S. L., Abrahamsen M. E., Giuliano A. R. (2021). Incidence and risk factors of C. trachomatis and N. gonorrhoeae among young women from the Western cape, South Africa: the EVRI study. *PLoS One*.

[44] Gaffoor Z., Wand H., Street R. A., Abbai N., Ramjee G. (2016). Predictors of perceived male partner concurrency among women at risk for HIV and STI acquisition in Durban, South Africa. *AIDS Research andTherapy*.

[45] Abbai N. S., Nyirenda M., Naidoo S., Ramjee G. (2018). Prevalent herpes simplex virus-2 increases the risk of incident bacterial vaginosis in women from South Africa. *AIDS andBehavavior*.

