# Supplementary material for: Significant Associations between Chlamydia trachomatis and Neisseria gonorrhoeae Infections in Human Immunodeficiency Virus-Infected Pregnant Women
Source: Infect Dis Obstet Gynecol. 2022 Jun 17;2022:7930567. doi: 10.1155/2022/7930567 (PMC9232329; doi:10.1155/2022/7930567)
Supplement: Supplementary Materials — That shows the raw data of Ct values for samples testing positive for C. trachomatis and N. gonorrhoeae. [file 7930567.f1.docx]

**Supplementary Table 1: Raw data of Ct values for samples testing positive for *C. trachomatis* and *N. gonorrhoeae***

|  | **Ct mean values** | |
| --- | --- | --- |
|  | ***C. trachomatis*** | ***N. gonorrhoeae*** |
| **Sample number** |  |  |
| BN039 | 32,343 | - |
| BN042 | - | 27,122 |
| BN049 | 29,114 | - |
| BN051 | 27,630 | - |
| BN057 | 28,611 | - |
| BN065 | 28,261 | - |
| BN079 | 27,682 | - |
| BN085 | 32,001 | - |
| BN088 | 29,878 | - |
| BN089 | 29,960 | - |
| BN094 | 28,238 | - |
| BN098 | 27,550 | - |
| BN110 | 31,724 | - |
| BN112 | 26,536 | - |
| BN116 | 29,631 | - |
| BN117 | 28,264 | 29,934 |
| BN122 | 27,340 | - |
| BN128 | 25,583 | - |
| BN134 | 27,637 | - |
| BN135 | 26,378 | 27,940 |
| BN149 | 28,146 | - |
| BN170 | 23,985 | - |
| BN171 | 26,861 | - |
| BN172 | 27,751 | - |
| BN178 | 26,170 | - |
| BN192 | 25,675 | - |
| BN193 | - | 30,799 |
| BN214 | - | 34,494 |
| BN226 | 31,448 | - |
| BN243 | 29,595 | 29,584 |
| BN245 | 27,537 | - |
| BN249 | 27,706 | - |
| BN251 | - | 34,652 |
| BN259 | 29,305 | - |
| BN261 | 30,788 | 33,626 |
| BN269 | 27,746 | - |
| BN270 | 27,563 | - |
| BN274 | 29,963 | - |
| BN282 | 24,892 | - |
| BN286 | 28,513 | - |
| BN289 | 25,109 | - |
| BN292 | 26,545 | 35,232 |
| BN305 | 25,237 | - |
| BN309 | 20,107 | 30,632 |
| BN310 | 34,173 | - |
| BN311 | - | 34,619 |
| BN322 | 29,772 | - |
| BN335 | - | 32,630 |
| BN342 | 23,672 | - |
| BN360 | 28,704 | - |
| BN362 | - | 35,344 |
| BN363 | - | 25,877 |
| BN369 | 30,224 | - |
| BN374 | 25,875 | - |
| BN375 | - | 34,263 |
| BN377 | 27,327 | 29,354 |
